# Multimodal assessment of clinical and neural changes following multidisciplinary management in functional motor disorders

**DOI:** 10.1007/s00702-026-03254-5

**Published:** 2026-08-06

**Authors:** Marialuisa Gandolfi, Angela Sandri, Elisabetta Sarasso, Andrea Gardoni, Michela Russo, Silvia Basaia, Ilaria Antonella Di Vico, Roberto Erro, Serena Camozzi, Giulia Pedrotti, Melania Fasoli, Francesca Salaorni, Christian Geroin, Giancarlo Mansueto, Francesca Benedetta Pizzini, Marco Barillari, Matteo Francesco Lauriola, Mariachiara Tozzi, Francesca Rusciano, Anna Fratucello, Sofia Cuoco, Immacolata Carotenuto, Carlo Ricciardi, Francesco Amato, Claudia Vinciguerra, Annibale Botto, Daniele Romano, Marianna Amboni, Mirta Fiorio, Angela Marotta, Giovanna Maddalena Squintani, Sara Mariotto, Stefano Tamburin, Fabio Paio, Giuseppe De Biasi, Giuseppe Piscosquito, Elisa Canu, Paolo Barone, Massimo Filippi, Michele Tinazzi, Maria Teresa Pellecchia, Federica Agosta

**Affiliations:** 1 Department of Neurosciences, Biomedicine and Movement Sciences, University of Verona, Pz.le LA Scuro, 10, 37134 Verona, Italy; 2 Azienda Ospedaliera Universitaria Integrata Verona, Verona, Italy; 3 Neuroimaging Research Unit, Division of Neuroscience, IRCCS San Raffaele Scientific Institute, Milan, Italy; 4 Neurotech Hub, Vita-Salute San Raffaele University, Milan, Italy; 5 Department of Neuroscience, Rehabilitation, Ophthalmology, Genetics and Maternal Child Health, University of Genoa, Genoa, Italy; 6 Department of Chemical, Material and Industrial Production Engineering, University of Naples Federico II, Naples, Italy; 7 Department of Medicine, Surgery and Dentistry “Scuola Medica Salernitana” University of Salerno, Salerno, Italy; 8 IRCCS Synlab SDN, Naples, Italy; 9 Department of Surgery, Dentistry, Pediatrics, and Gynecology, University of Verona, Verona, Italy; 10 Department of Diagnostic and Public Health, Section of Radiology, University of Verona, Verona, Italy; 11 Department of Radiology, Ospedale G.B. Rossi AOUI Verona, Verona, Italy; 12 Department of Electrical Engineering and Information Technologies, University of Naples Federico II, Naples, Italy; 13 Neurology Unit, University Hospital “San Giovanni di Dio e Ruggi d’Aragona”, Salerno, Italy; 14 UOC Neuroradiologia Diagnostica ed Interventistica Azienda Ospedaliera-Universitaria “San Giovanni di Dio e Ruggi d’Aragona”, Salerno, Italy; 15 Neurology Unit, IRCCS San Raffaele Scientific Institute, Milan, Italy; 16 Neurorehabilitation Unit, IRCCS San Raffaele Scientific Institute, Milan, Italy; 17 Neurophysiology service, IRCCS San Raffaele Scientific Institute, Milan, Italy; 18 Vita-Salute San Raffaele University, Milan, Italy

**Keywords:** Functional neurological disorder, Exercise therapy, Functional neuroimaging, Biomarkers

## Abstract

Functional motor disorders (FMD) are common and disabling conditions, but objective biomarker changes following multidisciplinary management remain limited. Our objective was to investigate changes and responsiveness of multimodal biomarkers in individuals with FMD undergoing multidisciplinary management. 34 patients with clinically definite FMD (mean age 42.76 ± 11.01 years; 85.7% female; mean disease duration 0.64 ± 1.52 years) underwent a standardized multimodal assessment at baseline and 3-month follow-up after a multidisciplinary program. The intervention included a 5-day intensive rehabilitation program (2 h/day), followed by a 12-week phase of telemedicine (one session every 2 weeks) and home-based self-management (two sessions/week). Multimodal biomarkers across motor, exteroceptive, interoceptive, and brain function domains were assessed using clinical, neurophysiological, and task-based functional MRI measures. Motor symptoms improved with concurrent gains in depressive symptoms, physical quality of life, and fatigue (all, *p* < 0.047). Specific improvements were observed in motor performance, including reduced stride time variability during cognitive dual-tasking (*p* = 0.018) and enhanced postural stability mainly in the eyes-closed condition (all, *p* < 0.028), while neurophysiological and nociceptive measures remained unchanged. Patients showed altered baseline brain activity compared with controls; post-intervention changes were observed in motor, cerebellar, and fronto-insular regions (*p* < 0.001, uncorrected), without correlations with clinical outcomes. Multidisciplinary management in FMD was associated with improved clinical outcomes and specific motor and neural changes, suggesting domain-specific responsiveness and a potential role for multimodal biomarkers, requiring confirmation in larger controlled studies. Trial Registration number NCT06328790. Registered on 26 March 2024.

**Supplementary Information:**

The online version contains supplementary material available at https://doi.org/10.1007/s00702-026-03254-5.

## Introduction

Functional Motor Disorders (FMD) are common and disabling conditions within the broader spectrum of Functional Neurological Disorders (FND) characterised by motor symptoms such as limb weakness, tremor, dystonia, gait disorders, often associated with non-motor symptoms, including pain and fatigue that further impact quality of life (Věchetová et al. 2018). The prevalence of FMD is high among patients attending neurology units, both as isolated and combined phenotypes (Stone et al. 2010; Tinazzi et al. 2020). This clinical complexity reinforces the need for refined therapeutic approaches.

FMD arises from a dysfunction of the neural system integrating interoceptive, exteroceptive, and motor signals, in which symptoms reflect altered perceptual inferences about the state of the body rather than structural damage (Hallett et al. 2022; Gandolfi et al. 2026). This model suggests shared mechanisms involving altered attention, sense of agency, and belief processing, underlying different symptoms presentations (Hallett et al. 2022; Gandolfi et al. 2026).

Early diagnosis based on positive clinical signs (i.e., Hoover sign) and identification of incongruence and inconsistency of symptoms remains a cornerstone of multidisciplinary management (Espay et al. 2018) Specialised rehabilitation within the biopsychosocial framework represents a key component (Gelauff et al. 2014; Nielsen et al. 2015; Gandolfi et al. 2025). Physiotherapy includes education, retraining of movement patterns, reduction of maladaptive attention to symptoms and restoration of motor control through motor relearning and behavioral change (Nielsen et al. 2015). Treatment response is variable, and mechanisms underlaying recovery are not fully understood. In this context, biomarkers may improve diagnostic precision and prognostic stratification (Thomsen et al. 2020).

Despite increasing interest, only a limited number of biomarkers have been validated in FMD and evidence is mainly cross-sectional and focused on single domains (Thomsen et al. 2020). Neurophysiological findings support diagnosis, mainly in functional tremor and myoclonus (Edwards et al. 2024). Neuroimaging studies have identified potential biomarkers not restricted to specific phenotypes consisting of hyperconnectivity across sensorimotor, attentional, and emotional networks (Stone et al. 2007; Voon et al. 2010; Schrag et al. 2013a, b; Maurer et al. 2016; Hassa et al. 2017; Nahab et al. 2017; Wegrzyk et al. 2018; Bègue et al. 2018; Thomsen et al. 2020). However, responsiveness to treatment remains poorly explored, and longitudinal multimodal studies are lacking (Thomsen et al. 2020).

This study explores changes in multimodal biomarkers following multidisciplinary management in individuals with FMD, reflecting motor, exteroceptive, and brain activity dysfunction. (Gandolfi et al. 2024) We hypothesized that a subset of these biomarkers, aligned with the underlying pathophysiology, may represent candidates sensitive to treatment-related changes in specific domains, warranting further validation.

## Methods

We conducted a prospective longitudinal study including 34 patients with a clinically definite diagnosis of FMD recruited at Neurological Unit B, AOUI Verona, Italy (Gupta and Lang 2009). Participants underwent a standardized multimodal assessment at baseline (T0) and at 3-month follow-up (T1) after a specialised multidisciplinary rehabilitation program (Gandolfi et al. 2024). Ethics approval was obtained (4201CESC-PNRR-MAD-2022-12376826), and all participants signed written informed consent.

Participants were eligible if they had been previously enrolled in the multimodal assessment study, met the predefined selection criteria outlined in the published study protocol, and completed the multidisciplinary program (Gandolfi et al. 2026). Healthy controls included in the imaging analyses were recruited consecutively according to the same eligibility criteria specified in the study protocol and underwent identical baseline assessment procedures, except for the longitudinal rehabilitation program (Gandolfi et al. 2024). Exclusion criteria were a Mini-Mental State Examination score < 24/30; physical conditions precluding the provision of informed consent; documented major neurological or major psychiatric disorders that could interfere with study participation or data interpretation (including neuropathy, seizures, major depressive disorder, ADHD, or autism spectrum disorder), (Gandolfi et al. 2024); and contraindications to 3T MRI.

Clinical assessment.

Demographic (age, sex) and clinical data (disease duration, phenotype) were collected according to protocol (Gandolfi et al. 2024). Motor symptom severity was assessed using the clinician-rated Simplified Functional Movement Disorders Rating Scale (S-FMDRS; range 0–54, higher scores indicate greater severity). (Nielsen et al. 2017) Fatigue, pain, anxiety, depression, and alexithymia were assessed using validated self-report instruments (Gandolfi et al. 2025): the Multidimensional Fatigue Inventory (MFI), the Brief Pain Inventory (BPI), the Beck Anxiety Inventory (BAI), the Beck Depression Inventory-II (BDI-II), and the Toronto Alexithymia Scale (TAS-20) (Beck A. T. 1993; Smets et al. 1995; Beck A. T., Beck, A. T., Steer, R. A., & Brown 1996; Bressi et al. 1996; Poquet and Lin 2016). Quality of life was assessed using the 12-item Short Form Health Survey (SF-12), including the physical and mental component summary scores (Gray et al. 2006). The Clinical Global Impression–Improvement (CGI-I) scale was used to assess perceived clinical change on a 7-point scale (1 = very much improved; 7 = very much worse) (Guy, 1976).

### Biomarkers assessment

Multimodal biomarkers across motor, exteroceptive, and cerebral domains were collected at T0 and T1 using a previously published standardised protocol (Vinciguerra et al. 2026; Gandolfi et al. 2026). This framework supports the development of diagnostic machine-learning algorithm based on multimodal biomarkers. (Gandolfi et al. 2026) In this longitudinal analysis, we selected a priori biomarkers to assess their responsiveness to the multidisciplinary intervention, including motor, neurophysiological, and MRI measurements (Gandolfi et al. 2026).

In the motor domain, we assessed blink reflex R2 response areas, spatiotemporal gait parameters, and postural control using instrumental gait analysis and stabilometry (Gandolfi et al. 2021, 2023a, 2026; Sandri et al. 2024; Vinciguerra et al. 2026). Blink reflex R2 areas were obtained from ipsilateral and contralateral responses following supraorbital nerve stimulation (square-wave pulses, 0.2 ms duration, supramaximal intensity) (Vinciguerra et al. 2026). Gait and postural parameters were evaluated under single- and dual-task conditions (Gandolfi et al. 2021, 2023a, 2026; Sandri et al. 2024). The single-task condition consisted of standing on the platform while fixating a cross at eye level and walking at a self-selected speed along a 10-m corridor (Gandolfi et al. 2021, 2023a, b; Sandri et al. 2024). The motor dual-task consisted of repetitive pronation–supination movements of the dominant hand, whereas the cognitive dual-task consisted of serial subtraction of seven from 100 (Gandolfi et al. 2021, 2023a, b; Sandri et al. 2024). A visual dual-task consisted of fixation on a target at eye level during gait. (Gandolfi et al. 2023a; Sandri et al. 2024) Spatiotemporal parameters included stride length (m), stride time (ms), gait speed (m/s), and stride time variability (Gandolfi et al. 2021, 2023a, 2026; Sandri et al. 2024). Postural control parameters included sway area (mm²), sway perimeter (mm), and center-of-pressure displacement in the anterior–posterior and medio–lateral directions under eyes-open and eyes-closed conditions (Gandolfi et al. 2021, 2023a, 2026; Sandri et al. 2024). Romberg ratios were calculated for the sway area. The Dual-Task Effect (DTE) was expressed as percentage changes relative to single-task performance (Plummer et al. 2015; Gandolfi et al. 2026).

In the exteroceptive domain, N2–P2 amplitude from laser-evoked potentials (Squintani et al. 2021; Gandolfi et al. 2026) were recorded from the foot during a conditioned pain modulation paradigm.

In the cerebral domain, two functional MRI (fMRI) tasks were used to assess brain activity in FMD patients using a 3T MRI scanner, a “motor-task” and a “dual-task” as previously proposed (Sarasso et al. 2021). The “motor-task” consisted of alternated self-paced dorsal and plantar flexion movements of the feet with eyes closed, whereas the “dual-task” involved the same antiphase foot movement executed while mentally counting backward by threes starting from 100. For both fMRI tasks, a block design (ABAB) was used, in which the activation A (lasting about 19 seconds) corresponded to foot movements, while during the resting period B (lasting about 19 seconds) subjects maintained eyes closed. Each period was repeated 6 times (total duration 3’47”). Tasks were performed in response to auditory stimuli (“go” and “stop”) with prior training outside the scanner and visual monitored by an observer during scanning to ensure correct execution.

Structural imaging (T1-, T2-, and fluid-attenuated inversion recovery - FLAIR) was performed to exclude relevant CNS lesions. MRI acquisition parameters and preprocessing procedures are detailed in the Supplementary Material. MRI scans were available at baseline for 63 subjects with FMD and 17 age- and sex-matched healthy controls. Healthy controls were consecutively recruited within the framework of the ongoing multimodal biomarker project according to the published study protocol (Gandolfi et al. 2024) They underwent the same baseline clinical, neurophysiological, and MRI assessment protocol as patients, including neurological history, Mini-Mental State Examination screening, and structural MRI to exclude relevant neurological disorders. Healthy controls participated exclusively in the baseline cross-sectional comparisons and did not undergo the multidisciplinary intervention or longitudinal follow-up assessments.

### Multidisciplinary program

FMD multidisciplinary management is a three-stage process: a 5-day intensive rehabilitation program disrupts maladaptive sensorimotor and attentional priors, telemedicine reinforces adaptive positive behaviour through iterative feedback, and self-management consolidates these changes into internally regulated control of movement and behaviour.

**Fig. 1:**
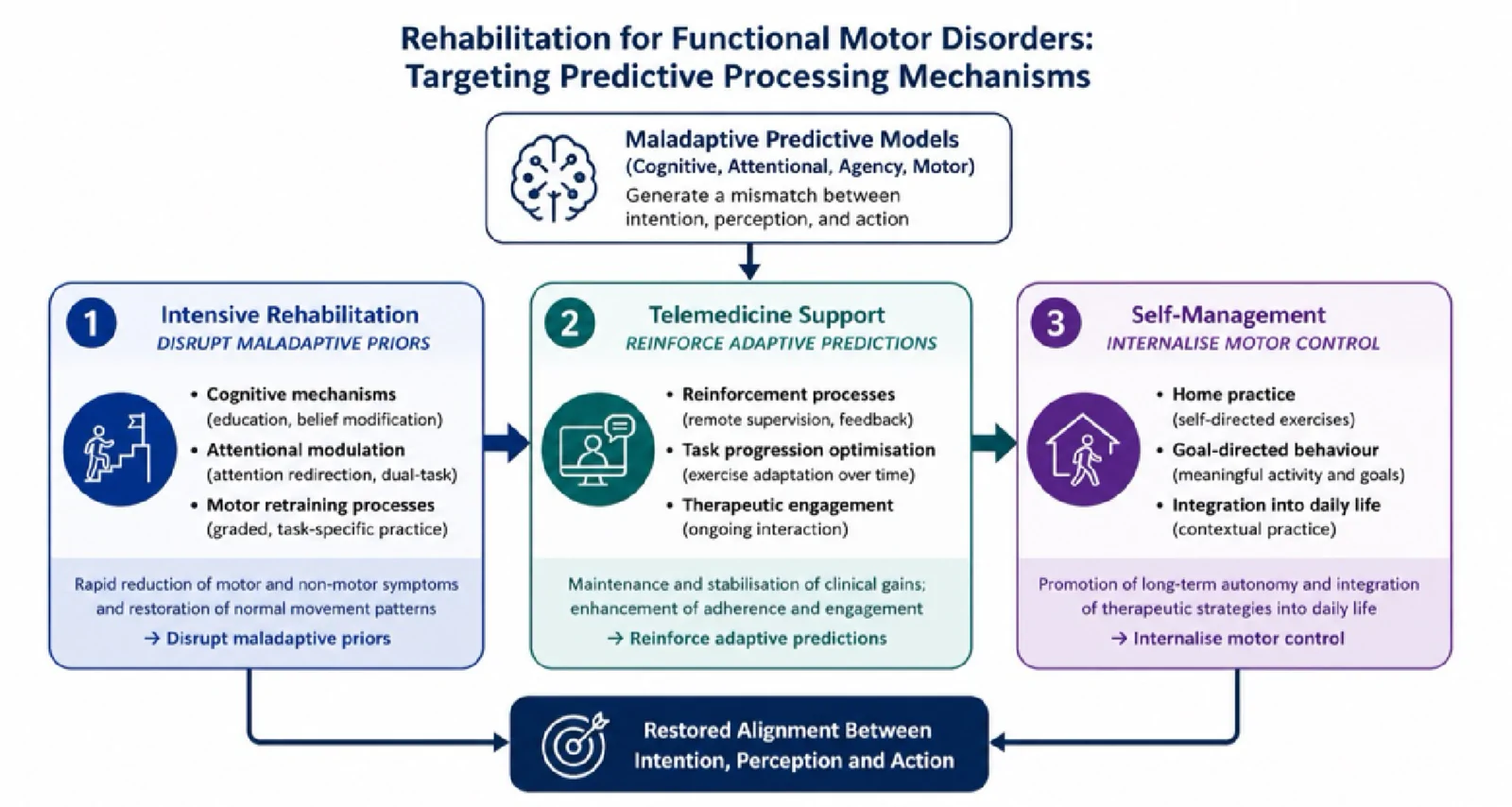
Staged management framework for functional motor disorders. Intensive rehabilitation disrupts maladaptive predictive models through education, attentional redirection, and motor retraining, promoting faster improvements. Telemedicine reinforces adaptive predictions and maintains clinical improvements. Self-management supports long-term autonomy and internalization of motor control. These three stages promote the restoration of alignment between intention, perception, and action. Figure created with the assistance of artificial intelligence (ChatGPT/OpenAI)

All patients completed a 5-day intensive rehabilitation program (2 h/day, 5 days) followed by a 12-week program including telemedicine sessions (every 2 weeks) and home-based sessions (1 h/session, 2 sessions/week). The conceptual framework is shown in Fig. 1, and intervention components are detailed in the Supplementary Material Tables 1 and 2.

### Statistical analysis

Descriptive statistics included mean (SD) or median (IQR) for continuous variables and counts and percentages for categorical variables. Within-group changes from T0 to T1 were assessed using paired t tests or Wilcoxon signed-rank tests, depending on data distribution. Normality was evaluated using the Shapiro–Wilk test. Mean differences (T1–T0) with 95% confidence intervals were estimated, including bootstrap resampling. Changes of approximately 5 points for the BDI-II and 3–5 points for the SF-12 physical component score were considered clinically meaningful (MCID) (Button et al. 2015; Díaz-Arribas et al. 2017).

### fMRI preprocessing and analysis

Before statistical analysis, images were realigned to the first volume to correct for motion (all participants showed maximal head movements lower than 3 mm in each direction), slice-timing corrected, coregistered to the subject’s 3DT1, spatially normalized into the standard MNI (Montreal Neurological Institute) space, and smoothed applying an 8-mm 3-D Gaussian filter. BOLD signal changes associated with the foot movement tasks (including movement parameters as confounds) were evaluated voxel by voxel using the General Linear Model (GLM) and Gaussian field theory. Specific effects were tested by applying appropriate linear contrasts. Significant hemodynamic changes for each contrast were evaluated using SPM.

Differences between groups (healthy controls vs. FMD) in each task were assessed using a two-sample t-test. Changes over time within each task and each group, were evaluated using a paired t-test. Between-group findings are FWE-corrected and shown at *p* < 0.05; longitudinal within-group findings are shown at *p* < 0.001 uncorrected; and only clusters greater than 10 voxels were considered. Mean BOLD signal was extracted from clusters showing significant differences after training in FMD subjects, and correlations with significant changes in clinical, gait analysis, postural control, and neurophysiological parameters were assessed using Spearman’s correlation coefficient in SPSS.

## Results

Participants had a mean age of 42.76 ± 11.01 years, and were mainly female (*n* = 30, 85.7%). The mean disease duration was 0.64 ± 1.52 years. Demographic characteristics of subjects included in the fMRI analyses are reported in the Supplementary Material (Supplementary Tables 3–5). Longitudinal fMRI analyses were conducted in a subsample: 26 patients had fMRI motor-task data available at both time points, and 20 had fMRI dual-task data. Clinical characteristics and FMD phenotypes are reported in Table 1. The number of participants contributing to each analysis is reported in the Results and summarized in Supplementary Table 10.

**Table 1:** Clinical characteristics and symptom distribution

Variable	*n* (%)
FMD symptoms	
Weakness	22 (64.7)
Gait disorders	21 (61.8)
Tremor	15 (44.1)
Other	14 (41.2)
Dystonia	8 (23.5)
Myoclonus	5 (14.7)
Symptom distribution	
Isolated (1 symptom)	7 (20.6)
Combined (≥ 2 symptoms)	27 (79.4)
Gait phenotype (*n* = 21)	
Slow gait	8 (36.4)
Other gait disorders	6 (27.3)
Ice walking	2 (9.1)
Paraparetic gait	2 (9.1)
Astasia–abasia	2 (9.1)
Knee buckling	2 (9.1)
Non-neurological comorbidities	
≥ 1 comorbidity	18 (52.9)
No comorbidities	16 (47.1)
Type of comorbidities	
Endocrine–metabolic	6 (17.6)
Musculoskeletal	3 (8.8)
Psychiatric	2 (5.9)
Other (less frequent)	2 (5.9)
Gynaecological	1 (2.9)
Gastrointestinal	1 (2.9)
Neurological	0 (0.0)
Associated FND symptoms	
Visual disturbances	18 (52.9)
Sensory symptoms	15 (44.1)
Cognitive symptoms	14 (41.2)
Pain (fibromyalgia)	8 (23.5)
FS	7 (20.6)
IBS	4 (11.8)
Associated FND distribution	
Isolated (1 symptom)	9 (26.5)
Combined (≥ 2 symptoms)	19 (55.9)
None	6 (17.6)

Psychiatric comorbidities refer to historical psychiatric conditions (one history of panic attacks, 1 history of fluctuating depressive symptoms) that did not meet the predefined exclusion criteria for major psychiatric disorders and were not present in the last year before enrolment

*FMD*  functional motor disorder, *FS*  functional seizures, *IBS* irritable bowel syndrome

**Fig. 2:**
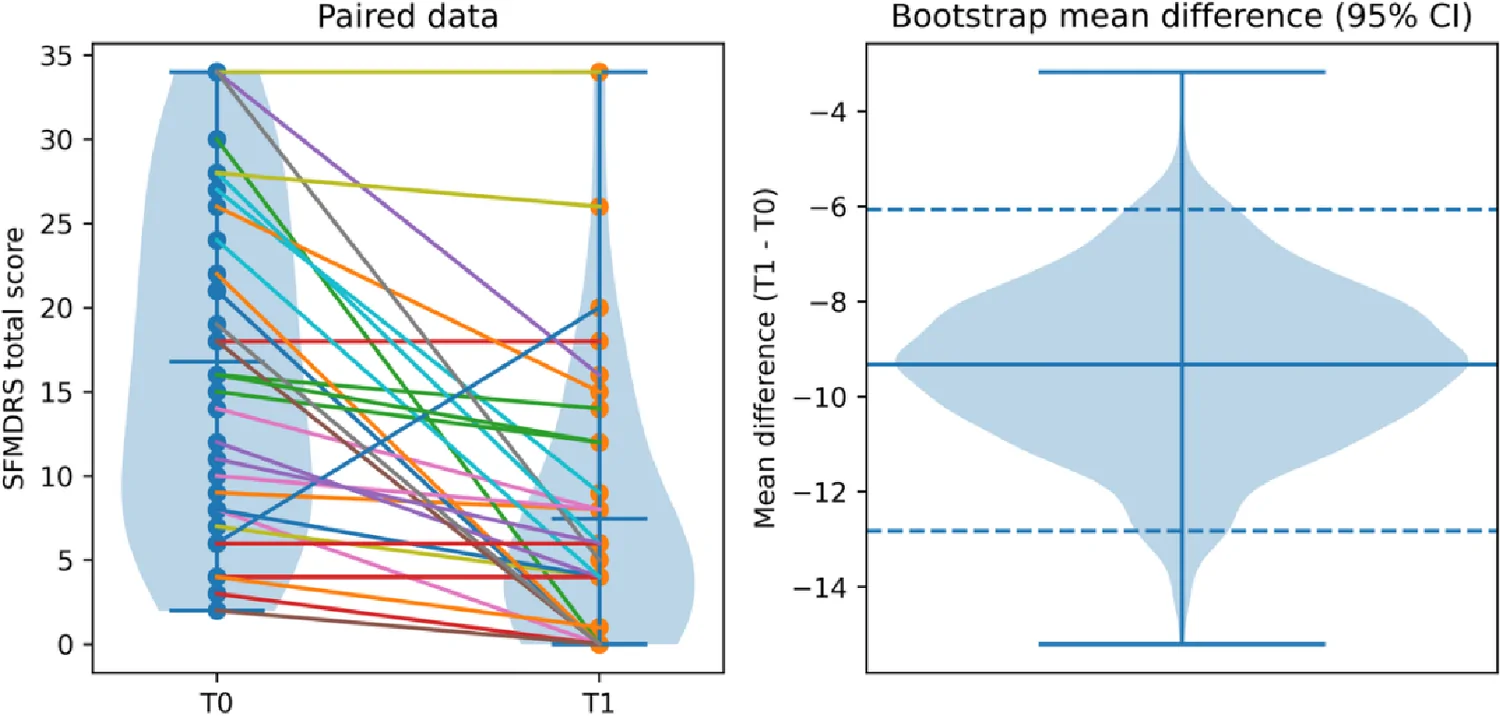
Changes in motor symptom severity at 3-month follow-up. Paired individual data showing S-FMDRS total scores at baseline (T0) and 3-month follow-up (T1). Each line represents an individual patient. Violin plots show the distribution of scores, with the mean overlaid. Bootstrap sampling distribution of the mean difference in S-FMDRS total score (T1–T0), with the mean difference and corresponding 95% confidence interval indicated. Negative values reflect an improvement in motor symptom severity

At 3-month follow-up, motor symptoms severity significantly improved by − 9.32 points on the S-FMDRS (Fig. 2) alongside reductions in anxiety, depressive symptoms (exceeding the MCID), fatigue, and improvements in physical Quality of life (exceeding the MCID). No significant changes were observed in the remaining outcomes. Statistics are reported in Table 2. Overall, 58.8% of patients (*n* = 20) were classified as improved, 20.6% (*n* = 7) showed no change, and 20.6% (*n* = 7) worsened at the CGI-I scale.

**Table 2:** Changes in clinical outcomes from baseline (T0) to 3-month follow-up (T1)

Variables	T0 (mean ± SD)	T1 (mean ± SD)	*p* value
S-FMDRS total score	16.79 ± 9.88	7.47 ± 8.10	< 0.001*
TAS-20 total score	48.24 ± 14.43	46.59 ± 16.90	0.050
BAI total score	18.56 ± 10.38	16.59 ± 10.07	0.047*
BDI-II total score	15.85 ± 11.24	10.35 ± 10.75	0.001*
BDI-II somatic subscore	10.50 ± 6.92	6.32 ± 5.85	< 0.001*
BDI-II cognitive subscore	5.35 ± 5.47	4.00 ± 5.91	0.059
Worst pain	5.44 ± 4.00	4.97 ± 3.75	0.228
Least pain	1.56 ± 2.49	1.53 ± 2.36	0.980
Average pain	4.09 ± 3.13	3.71 ± 3.08	0.402
Current pain	3.12 ± 3.23	2.56 ± 2.84	0.105
BPI mean interference score	3.57 ± 3.04	3.04 ± 2.85	0.113
SF-12 physical component score	33.19 ± 10.55	38.20 ± 11.19	0.003*
SF-12 mental component score	42.60 ± 9.88	43.28 ± 10.26	0.533
MFI-20 general fatigue	15.09 ± 3.60	14.44 ± 2.20	0.169
MFI-20 physical fatigue	14.18 ± 3.68	9.38 ± 2.50	< 0.001*
MFI-20 reduced activity	12.29 ± 4.78	10.15 ± 2.68	0.007*
MFI-20 reduced motivation	9.50 ± 3.17	7.53 ± 1.81	0.001*
MFI-20 mental fatigue	11.32 ± 4.10	11.53 ± 2.08	0.702
MFI-20 total score	62.38 ± 14.69	53.03 ± 8.99	< 0.001*
BAQ total score	77.94 ± 18.81	76.97 ± 21.27	0.955

*S-FMDRS *  simplified functional movement disorders rating scale, *TAS-20* Toronto alexithymia scale, *BAI* beck anxiety inventory, *BDI* beck depression inventory-II, *BPI* brief pain inventory, *SF-12* 12-item short form health survey, *MFI-20* multidimensional fatigue inventory, *BAQ*  body awareness questionnaire

*p < 0.05

In the motor domain, a significant reduction in stride time variability was reported during the cognitive dual-task condition (*p* = 0.018), indicating improved gait stability under cognitive load. Postural control improved particularly in the eye-closed condition during single-task, with reduction in sway area, perimeter, and velocity of the CoP displacement in both anteroposterior and mediolateral directions, as well as in eyes-open perimeter. A significant improvement in DTE in the sway area was observed during the cognitive dual-task condition. Neurophysiological and nociceptive measures did not show significant changes. For details see Supplementary Tables 6–8.

### Imaging results

**Fig. 3:**
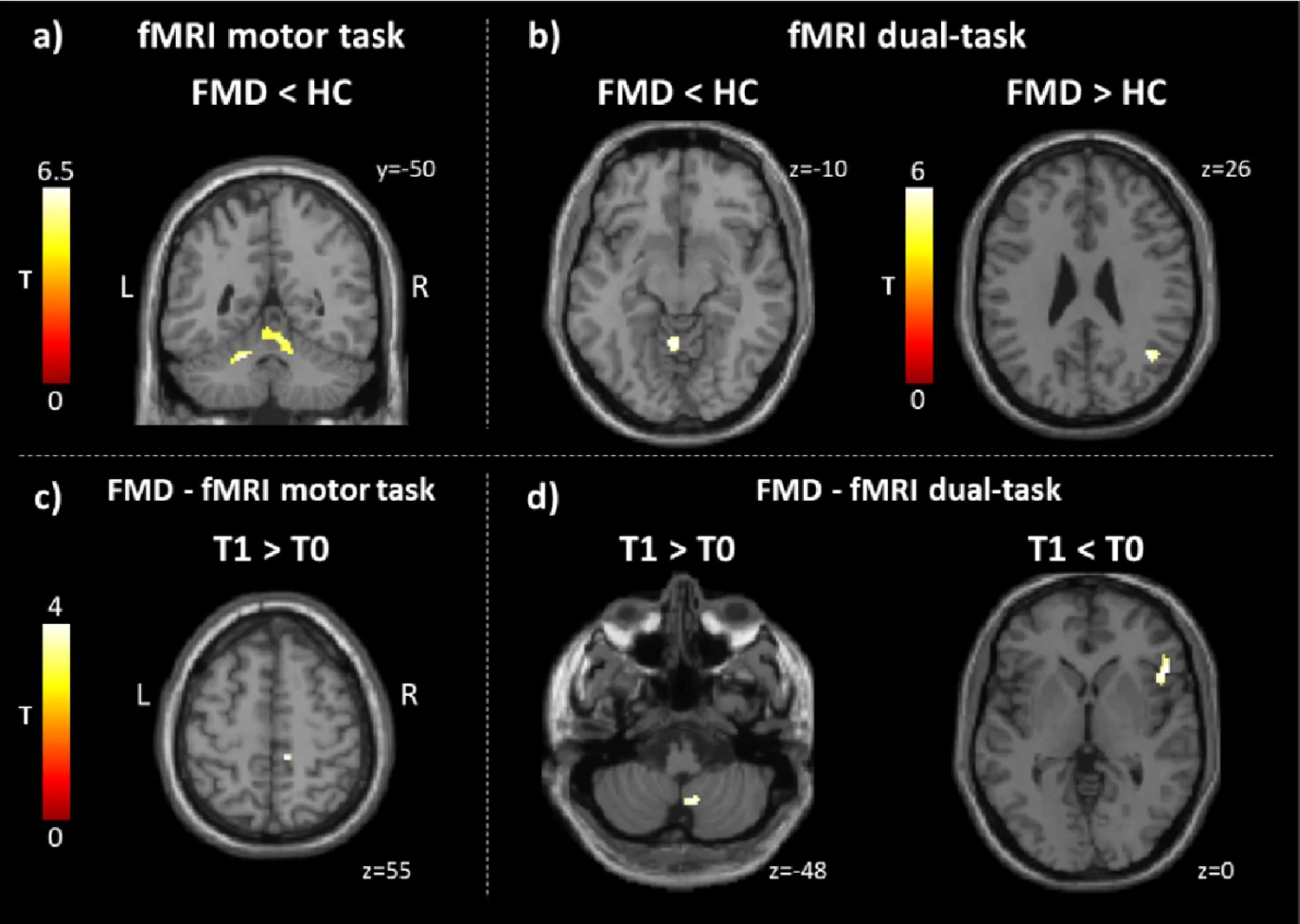
**a** Brain activity during fMRI motor-task (**a**) and dual-task (**b**) in FMD patients relative to healthy controls. Longitudinal changes in brain activity during fMRI motor task (**c**) and dual-task (**d**) after rehabilitation in patients with FMD. Findings in panels (**a**) and (**b**) are shown at *p* < 0.05 FWE corrected; findings in panels (**c**) and (**d**) are shown at *p* < 0.001 uncorrected. Only clusters greater than 10 voxels are reported. Results are shown on axial sections of the Montreal Neurological Institute standard brain. Color bars denote T values. Abbreviations: FMD = Functional Movement Disorders; fMRI = functional magnetic resonance imaging; HC = healthy controls; T0 = baseline (pre-rehabilitation); T1 = post-rehabilitation

At baseline, patients with FMD showed reduced activity of bilateral cerebellum IV-V and vermis IV-V relative to healthy controls during the fMRI motor task, while they showed reduced activity of left cerebellum lobule IV-V and vermis IV-V, and increased activity of right angular gyrus during the fMRI dual-task (Fig. 3a and b and Supplementary Table 5). After training, patients with FMD showed increased activity in the right paracentral lobule during the fMRI motor task; moreover, they showed increased activity in the right cerebellum IX and reduced activity in the right inferior frontal gyrus pars opercularis/insula during the fMRI dual-task (Fig. 3c and d and Supplementary Table 9). No correlations were found between fMRI changes and clinical improvements in FMD.

## Discussion

Our findings suggest that only a subset of the investigated biomarkers showed sensitivity to change following multidisciplinary management, particularly those related to motor control and attentional modulation, including gait variability under cognitive dual-task conditions, postural stability parameters in the eyes - closed condition, and task-related brain activity. Neurophysiological and nociceptive measures remained unchanged. This pattern suggests a domain-specific responsiveness rather than global responsiveness of biomarker in FMD.

The observed improvements in motor performance and postural control suggest that multidisciplinary rehabilitation may target mechanisms underlying motor control and attentional process (Gelauff et al. 2014). Particularly relevant is the finding that postural stability parameters improved in the eyes-closed condition, where internal focus of attention is typically emphasized and, in FMD, usually induces worsening of performance (Woollacott and Shumway-Cook 2002). This improvement suggests that FMD specialised rehabilitation might enhance postural stability even in this challenging condition, in which visual information is missing, and patients may rely more efficiently on sensorimotor integration processes (Woollacott and Shumway-Cook 2002).

Neuroimaging findings provide preliminary insight into the neural mechanisms that may accompany these domain-specific changes. We investigated task-related brain activity in FMD patients during two fMRI tasks: a motor task and a cognitive dual-task. Patients showed altered brain activity relative to baseline healthy controls activity during both tasks, with reduced recruitment of cerebellar lobules IV and V, mainly during the fMRI motor task, and increased activity of the angular gyrus during the fMRI dual-task. Cerebellar areas IV-V are sensorimotor regions (Guell and Schmahmann 2020) and their reduced activity may reflect a disrupted sensorimotor-cerebellar network in the context of altered motor control and sense of agency in FMD (Sasikumar and Strafella 2021). The increased activity in the angular gyrus, involved in attention reorientation and problem solving (Seghier 2013), during fMRI dual-task in FMD patients might reflect a reallocation of attentional resources away from excessive self-focused motor monitoring during the dual-task. ^53–55^ These findings further support the use of dual-task training in FMD rehabilitation.

Finally, fMRI tasks revealed neural changes following rehabilitation. After the intervention, patients showed increased activity in the paracentral gyrus during motor task, which plays a crucial role in voluntary control of the lower limb and foot, possibly suggesting partial restoration of motor control. During the fMRI dual-task, increased activity in a cerebellar area involved in attentive processes (Guell and Schmahmann 2020) and decreased activity in the inferior frontal gyrus and insula were observed, regions involved in the aberrant emotional processes that typically interfere with movement in FMD (Roelofs et al. 2019; Perez et al. 2021).

Evidence on rehabilitation in FMD is limited to a few adequately powered randomized controlled trials available (Dallocchio et al. 2016; Nielsen et al. 2024; Gandolfi et al. 2025). These studies differed from each other and from our study in terms of the nature (Cognitive Behavioral Therapy associated with adapted physical activity program) and intensity of intervention (nine specialised physiotherapy sessions delivered over 3 weeks) (Dallocchio et al. 2015; Nielsen et al. 2024). Moreover, they relied on subjective (self-reported) outcomes (Dallocchio et al. 2015; Nielsen et al. 2024). No previous studies have investigated combined behavioral and MRI changes following multidisciplinary management in FMD, limiting direct comparisons with existing literature. These results align with our previous single-blind randomised controlled trial demonstrating the cost-effectiveness of a multidisciplinary approach including specialised physiotherapy for FMD supported by telemedicine in both objective (S-FMDRS) and subjective (QoL) measures (Gandolfi et al. 2025), extending current knowledge on specialized rehabilitation-related mechanisms in FMD (Gandolfi et al. 2025). Clinical improvement was not paralleled by changes across all biomarker domains, suggesting differential responsiveness of these measures to short-term rehabilitation. The stability of neurophysiological measures should not necessarily be interpreted as a negative finding but may reflect lower responsiveness to short-term rehabilitation. Although current evidence supports shared pathophysiological mechanisms across FMD phenotypes (Hallet et al. 2022), the clinical heterogeneity of our cohort may have contributed to the variability observed in treatment response. This interpretation requires confirmation in larger controlled studies.

Beyond their mechanistic relevance, these findings may also inform the design of future randomized controlled trials in FMD. Motor performance measures may represent promising objective outcomes for monitoring treatment response, whereas neurophysiological and MRI measures may be more informative for investigating treatment mechanisms. Larger controlled studies are needed to validate these preliminary observations.

Some limitations should be acknowledged. The lack of a control group prevents conclusions about intervention effectiveness. Although a priori sample size estimation supported the study, the relatively small subset of patients undergoing pre–post fMRI task assessment limits statistical power. Additionally, phenotype-specific analyses were not performed because most participants presented combined rather than isolated motor phenotypes, limiting subgroup comparisons. Future larger multicentre studies should specifically investigate whether biomarker responsiveness differs across clinical phenotypes. Finally, the longitudinal fMRI analyses were exploratory in nature and did not survive correction for multiple comparisons. Therefore, these findings cannot be considered biomarkers of treatment response. Instead, they should be interpreted with caution as preliminary mechanistic insights and require validation in larger, adequately powered cohorts. Future studies should also examine their association with clinical outcomes to facilitate a more robust interpretation of their potential relevance.

A key strength of the study is the relatively large cross-sectional sample, enabling identification of robust findings that remained significant after family-wise error correction. Another strength is the integration of standardized clinical and functional MRI measures, enabling a multimodal evaluation of neural and clinical changes. Nonetheless, findings remain preliminary and warrant confirmation in larger controlled cohorts.

## Conclusion

Specialized rehabilitation resulted in domain-specific clinical improvements and changes in imaging measures related to motor control and attentional processes, supporting further investigation in FMD. The observed imaging findings should be considered preliminary mechanistic observations requiring confirmation in larger controlled studies.

## Supplementary Information

Below is the link to the electronic supplementary material.

Supplementary Material 1

## Data Availability

The datasets used and/or analyzed during the current study are available from the corresponding author on reasonable request.
