# Supplementary material for: Multimodal assessment of clinical and neural changes following multidisciplinary management in functional motor disorders
Source: J Neural Transm (Vienna). 2026 Aug 6;133(9):2733–43. doi: 10.1007/s00702-026-03254-5 (PMC13687378; doi:10.1007/s00702-026-03254-5)
Supplement: Supplementary file 1 [file 702_2026_3254_MOESM1_ESM.docx]

**SUPPLEMENTARY MATERIALS**

**Title**:

Multimodal assessment of clinical and neural changes following multidisciplinary management in functional motor disorders

Journal: Journal of Neural Transmission

Authors

Marialuisa Gandolfi ^1,2,^*

Angela Sandri ^2,^ *

Elisabetta Sarasso ^3,4,5^ *

Andrea Gardoni  ^3,4^

Michela Russo  ^6^

Silvia Basaia  ^3,4^

Massimo Filippi ^3,4,7,8,9,10^

Michele Tinazzi ^1,2^

Maria Teresa Pellecchia ^11,12^ °

Federica Agosta ^3,4,7,10^ °

BOdy Mind-Brain FMD Study Group

Authors Affiliations

1 Department of Neurosciences, Biomedicine and Movement Sciences, University of Verona, Verona, Italy,

2 Azienda Ospedaliera Universitaria Integrata Verona, Verona, Italy,

3 Neuroimaging Research Unit, Division of Neuroscience, IRCCS San Raffaele Scientific Institute, Milan, Italy,

4 Neurotech Hub, Vita-Salute San Raffaele University, Milan, Italy,

5 Department of Neuroscience, Rehabilitation, Ophthalmology, Genetics and Maternal Child Health, University of Genoa, Genoa, Italy.

6 Department of Chemical, Material and Industrial Production Engineering, University of Naples Federico II, Naples, Italy

7 Neurology Unit, IRCCS San Raffaele Scientific Institute, Milan, Italy,

8 Neurorehabilitation Unit, IRCCS San Raffaele Scientific Institute, Milan, Italy

9 Neurophysiology service, IRCCS San Raffaele Scientific Institute, Milan, Italy,

10 Vita-Salute San Raffaele University, Milan, Italy

11 Department of Medicine, Surgery and Dentistry "Scuola Medica Salernitana" University of Salerno, Italy

12 IRCCS Synlab SDN, Naples, Italy

* Contributed equally

° Contributed equally

**BOdy Mind-Brain FMD Study Group**

Ilaria Antonella Di Vico ^2^

Serena Camozzi ^1^

Christian Geroin ^13^

Giulia Pedrotti ^1^

Melania Fasoli ^1^

Francesca Salaorni ^1^

Giancarlo Mansueto  ^14,15^

Francesca Benedetta Pizzini ^15^

Marco Barillari  ^14^

Matteo Francesco Lauriola ^2^

Mariachiara Tozzi ^2^

Francesca Rusciano ^2^

Anna Fratucello ^2^

Roberto Erro ^11,12^

Sofia Cuoco ^11^

Immacolata Carotenuto ^11^

Carlo Ricciardi ^16^

Francesco Amato ^16^

Claudia Vinciguerra ^17^

Annibale Botto ^18^

Daniele Romano ^18^

Marianna Amboni ^19^

Mirta Fiorio ^1^

Angela Marotta ^1^

Giovanna Maddalena Squintani ^2^

Sara Mariotto ^2^

Stefano Tamburin ^1^

Fabio Paio ^1^

Giuseppe De Biasi ^11^

Giuseppe Piscosquito ^17^

Elisa Canu ^3,4^

Paolo Barone ^11,12^

**Affiliation study Group**

1 Department of Neurosciences, Biomedicine and Movement Sciences, University of Verona, Verona, Italy,

2 Azienda Ospedaliera Universitaria Integrata Verona, Verona, Italy,

13 Department of Surgery, Dentistry, Pediatrics, and Gynecology, University of Verona, Verona, Italy.

14 Department of Diagnostic and Public Health, Section of Radiology, University of Verona, P.le L.A. Scuro 10, 37134, Verona, Italy.

15 Department of Radiology, Ospedale G.B. Rossi AOUI Verona, Verona, Italy.

16 Department of Electrical Engineering and Information Technologies, University of Naples Federico II, Naples, Italy

17 Neurology Unit, University Hospital "San Giovanni di Dio e Ruggi d'Aragona, Salerno, Italy

18 UOC Neuroradiologia Diagnostica ed Interventistica Azienda Ospedaliera-Universitaria “San Giovanni di Dio e Ruggi d’Aragona”, Salerno

19 Department of Medicine, Center for Neurodegenerative Diseases (CEMAND), University of Salerno, Salerno, Italy.

Corresponding authors:

Michele Tinazzi; [michele.tinazzi@univr.it](mailto:michele.tinazzi@univr.it)

Department of Neurosciences, Biomedicine and Movement Sciences, University of Verona,

Pz.le LA Scuro, 10 – 37134 – Verona (Italy)

Marialuisa Gandolfi; [marialuisa.gandolfi@univr.it](mailto:marialuisa.gandolfi@univr.it)

Department of Neurosciences, Biomedicine and Movement Sciences, University of Verona,

Pz.le LA Scuro, 10 – 37134 – Verona (Italy

Supplementary Table 1. TIDieR-informed description of the 5-day in-person rehabilitation program

| TIDieR item | Description |
| --- | --- |
| Brief name | Intensive multidisciplinary rehabilitation program for functional motor disorders |
| Why | To disrupt maladaptive predictive models across cognitive, attentional, and motor domains |
| What | Education on diagnosis and symptom mechanisms; symptom–movement exploration using positive signs; attention redirection during functional tasks; task-specific motor retraining; sensory–motor integration (eg, mirror, video); avoidance of maladaptive compensatory strategies |
| Who provided | Multidisciplinary team including physiotherapists trained in functional motor disorders |
| How | Face-to-face, individual sessions with therapist guidance |
| Where | Outpatient rehabilitation setting |
| When and how much (dose) | Delivered over 5 consecutive days (2h/day); multiple daily sessions targeting different domains (motor, cognitive, behavioural) |
| Tailoring | Exercises individualised based on symptom type, severity, and functional limitations |
| Progression | Tasks progressively adapted by increasing speed, complexity, and dual-task demands, and reducing external support |
| Strategies to enhance adherence | Active patient engagement, goal setting, and use of feedback (verbal, visual) |
| Mechanistic rationale | Disruption of maladaptive priors and restoration of automatic motor control through prediction error updating |

Supplementary Table 2. TIDieR-informed description of the home-based self-management and telemedicine program.

| TIDieR item | Description |
| --- | --- |
| Brief name | Home-based self-management program supported by telemedicine |
| Why | To reinforce adaptive motor predictions and promote their internalisation into daily life |
| What | Home exercises targeting lower and upper limb function, gait and balance, dual-task performance, and symptom modulation (eg, tremor, dystonia); integration into daily activities |
| Who provided | Patient-led practice with remote support from the rehabilitation team |
| How | Home-based exercises supported by telemedicine (remote supervision, feedback, and adjustment of tasks) |
| Where | Patient’s home environment |
| When and how much (dose) | Daily practice integrated into routine activities; periodic telemedicine follow-up (frequency tailored) |
| Tailoring | Exercises adapted to individual symptoms, goals, and progression over time |
| Progression | Gradual increase in task complexity, variability, and dual-task demands |
| Strategies to enhance adherence | Goal setting, integration into meaningful activities, and ongoing therapeutic interaction via telemedicine |
| Mechanistic rationale | Reinforcement of adaptive predictions and internalisation of motor control through repetition, feedback, and behavioural integration |

**Methods**

Brain MRI scans were obtained using a 3.0 Tesla MRI scanner (Ingenia CX, Philips Medical Systems, Best, the Netherlands). Details of the acquired sequences are:

- 3D-T1: Repetition Time (TR) = 7 ms, Echo Time (TE) = 3.2 ms, flip angle = 9°, 204 contiguous sagittal sections, thickness = 1mm, Field of View (FOV) = 256mm x 240mm, matrix = 256x240, Voxel size 1x1x1;
- T2 echo planar imaging for fMRI tasks: TR = 1572ms; TE = 35 ms; flip angle = 70°; FOV = 240mm × 240mm; matrix = 96x94, voxel size = 2.5×2.5; slice thickness = 3 mm; 144 sets of 48 contiguous axial slices; acquisition time = 3’and 47”.

FMRI data were analyzed using SPM12 software (http://www.fil.ion.ucl.ac.uk/spm; Wellcome Trust Center for Neuroimaging, London, UK).

**Results**

**Supplementary table 3 -** Demographic characteristics of healthy controls and FMD patients with fMRI motor-task.

|  | **HC (N=17)** | **FMD (N=26)** | **p** |
| --- | --- | --- | --- |
| **Age (years)** | 47.88 ± 9.23 (31; 63) | 43.04 ± 10.23 (18; 62) | 0.09 |
| **Sex (M/F)** | 5/12 | 3/23 | 0.23 |

Values are mean ± standard deviation (min; max). p refers to Mann-Whitney test.

Abbreviations. FMD = functional movement disorders; HC = healthy controls; M/F = male/female; N = number.

**Supplementary table 4 -** Demographic characteristics of healthy controls and FMD patients with fMRI dual-task pre rehabilitation.

|  | **HC (N=17)** | **FMD (N=20)** | **p** |
| --- | --- | --- | --- |
| **Age (years)** | 47.88 ± 9.23 (31; 63) | 44.85 ± 10.40 (29; 63) | 0.27 |
| **Sex (M/F)** | 5/12 | 3/17 | 0.43 |

Values are mean ± standard deviation (min; max). p refers to Mann-Whitney test.

Abbreviations. FMD = functional movement disorders; HC = healthy controls; M/F = male/female; N = number.

**Supplementary table 5 -** Altered brain activity during fMRI tasks in FMD patients relative to healthy controls

| **fMRI MOTOR-TASK** | | | | | | |
| --- | --- | --- | --- | --- | --- | --- |
| **HC > FMD** | | | | | | |
| **Ke** | **T** | **BA** | **x** | **y** | **z** | **Area** |
| 131 | 6.56 | - | -16 | -46 | -26 | Left cerebellum IV-V |
| 114 | 5.65 | - | 10 | -48 | 18 | Right cerebellum IV-V |
|  | 5.12 | - | -2 | -50 | -12 | Vermis IV-V |
| **fMRI DUAL-TASK** | | | | | | |
| **HC > FMD** | | | | | | |
| **Ke** | **T** | **BA** | **x** | **y** | **z** | **Area** |
| 49 | 5.42 | - | -4 | -54 | -10 | Left/Vermis IV-V |
| **FMD > HC** | | | | | | |
| 51 | 5.79 |  | 42 | -58 | 26 | Right angular |

X, Y and Z coordinates refer to the Montreal Neurological Institute (MNI) space. Table reports results of the two-sample t-test performed in SPM (p < 0.05 FWE corrected). Only clusters greater than 10 voxels are reported.

fMRI motor-task consisted of antiphase feet dorsiflexion; fMRI dual-task consisted in the same feet movement while counting back by threes starting from 100. **Abbreviations:** BA = Brodmann Area; FMD = functional movement disorders; HC = healthy controls; Ke = cluster extension.

Supplementary Table 6. Spatiotemporal gait parameters across single- and dual-task conditions.

| **Variables** | **T0 (mean ± SD)** | **T1 (mean ± SD)** | **p value** |
| --- | --- | --- | --- |
| **Single task** |  |  |  |
| Stride length (m) | 1.16 ± 0.29 | 1.25 ± 0.35 | 0.546 |
| Stride time (ms) | 1173.30 ± 118.06 | 1171.48 ± 106.23 | 0.753 |
| Gait speed (m/s) | 0.96 ± 0.35 | 1.01 ± 0.32 | 0.658 |
| Stride time variability | 3.64 ± 2.12 | 2.64 ± 1.25 | 0.153 |
| **Motor dual-task** |  |  |  |
| Stride length (m) | 1.19 ± 0.23 | 1.14 ± 0.20 | 0.157 |
| Stride time (ms) | 1245.93 ± 160.59 | 1242.50 ± 136.33 | 0.451 |
| Gait speed (m/s) | 0.91 ± 0.31 | 0.93 ± 0.21 | 0.372 |
| Stride time variability | 4.62 ± 2.67 | 3.04 ± 1.54 | 0.068 |
| Dual-task effect (motor) speed | −5.83 ± 16.15 | −5.96 ± 14.40 | 0.273 |
| **Cognitive dual-task** |  |  |  |
| Stride length (m) | 1.16 ± 0.22 | 1.15 ± 0.20 | 0.904 |
| Stride time (ms) | 1230.11 ± 146.67 | 1324.60 ± 182.85 | 0.145 |
| Gait speed (m/s) | 0.87 ± 0.29 | 0.91 ± 0.22 | 0.837 |
| Stride time variability | 5.67 ± 3.75 | 3.54 ± 1.57 | 0.018* |
| **Visual dual-task** |  |  |  |
| Stride length (m) | 1.27 ± 0.18 | 1.23 ± 0.30 | 0.421 |
| Stride time (ms) | 1155.77 ± 118.32 | 1166.74 ± 91.56 | 0.071 |
| Gait speed (m/s) | 1.09 ± 0.22 | 1.06 ± 0.24 | 0.641 |
| Stride time variability | 3.55 ± 2.48 | 3.18 ± 1.94 | 0.852 |

Legend: SD = standard deviation; DTE = dual-task effect; *, p < 0.05.

Supplementary Table 7. Postural sway parameters across single- and dual-task conditions.

| **Variables** | **T0 (mean ± SD)** | **T1 (mean ± SD)** | **p value** |
| --- | --- | --- | --- |
| **Single task** |  |  |  |
| EO sway area (mm²) | 383.52 ± 253.26 | 356.57 ± 238.63 | 0.325 |
| EO perimeter (mm) | 343.22 ± 133.79 | 285.67 ± 97.00 | 0.023* |
| EO A/P (mm) | 5.23 ± 2.57 | 5.50 ± 1.98 | 0.682 |
| EO M/L (mm) | 4.07 ± 1.65 | 3.58 ± 1.53 | 0.190 |
| EC sway area (mm²) | 1752.84 ± 1508.02 | 920.22 ± 701.66 | 0.007* |
| EC perimeter (mm) | 647.48 ± 275.65 | 502.60 ± 178.12 | 0.005* |
| EC A/P (mm) | 9.88 ± 5.08 | 8.11 ± 4.26 | 0.028* |
| EC M/L (mm) | 8.73 ± 4.50 | 6.41 ± 3.01 | 0.012* |
| Romberg SA ST (EC/EO) ×100 | 300.87 ± 224.67 | 261.84 ± 155.34 | 0.792 |
| **Motor dual-task** |  |  |  |
| EO sway area (mm²) | 343.48 ± 243.31 | 284.49 ± 159.40 | 0.493 |
| EO perimeter (mm) | 498.12 ± 179.05 | 481.40 ± 168.12 | 0.568 |
| EO A/P (mm) | 4.18 ± 1.48 | 4.25 ± 1.53 | 0.271 |
| EO M/L (mm) | 4.03 ± 1.50 | 4.15 ± 1.70 | 0.581 |
| EC sway area (mm²) | 670.82 ± 470.90 | 552.69 ± 391.04 | 0.305 |
| EC perimeter (mm) | 817.45 ± 432.56 | 597.85 ± 189.09 | 0.112 |
| EC A/P (mm) | 6.95 ± 3.24 | 6.20 ± 2.81 | 0.456 |
| EC M/L (mm) | 6.74 ± 2.94 | 5.30 ± 1.88 | 0.052 |
| Romberg SA DTM (EC/EO) ×100 | 201.32 ± 91.49 | 177.29 ± 70.02 | 0.443 |
| DTE motor EC area | −3.27 ± 63.73 | −2.24 ± 67.99 | 0.940 |
| **Cognitive dual-task** |  |  |  |
| EO sway area (mm²) | 421.51 ± 336.87 | 418.30 ± 310.25 | 0.350 |
| EO perimeter (mm) | 391.70 ± 206.71 | 391.63 ± 175.51 | 0.922 |
| EO A/P (mm) | 5.81 ± 3.35 | 5.52 ± 2.96 | 0.713 |
| EO M/L (mm) | 4.16 ± 1.55 | 3.67 ± 1.33 | 0.054 |
| EC sway area (mm²) | 537.21 ± 414.42 | 516.07 ± 446.54 | 0.670 |
| EC perimeter (mm) | 484.67 ± 200.46 | 508.85 ± 217.92 | 0.387 |
| EC A/P (mm) | 6.56 ± 3.12 | 6.96 ± 4.00 | 0.767 |
| EC M/L (mm) | 5.31 ± 2.59 | 4.99 ± 2.38 | 0.478 |
| Romberg SA DTC (EC/EO) ×100 | 132.00 ± 56.65 | 174.77 ± 101.27 | 0.140 |
| DTE cognitive EC area | −39.84 ± 42.20 | −19.15 ± 55.93 | 0.037* |

Legend: SD = standard deviation; EO = eyes open; EC = eyes closed; SA = sway area; A/P = anteroposterior; M/L = mediolateral; CoP = centre of pressure; DTE = dual-task effect; *, p < 0.05. Romberg ratios were calculated as the ratio between EC and EC/EO conditions, expressed as a percentage. No significant changes were detected in laser-evoked potentials, pain ratings, DNIC-related measures, or blink reflex parameters (Supplementary Table 5).

Supplementary Table 8. Neurophysiological and sensory parameters at baseline and follow-up.

| Variables | T0 (mean ± SD) | T1 (mean ± SD) | p value |
| --- | --- | --- | --- |
| Lower Limb sensory threshold (J) | 1.29 ± 0.29 | 1.28 ± 0.37 | 0.353 |
| Lower Limb stimulus intensity | 2.55 ± 0.66 | 2.40 ± 0.32 | 0.426 |
| Lower Limb baseline N2–P2 amplitude (µV) | 23.76 ± 9.87 | 21.67 ± 6.87 | 0.773 |
| Lower Limb baseline N2–P2 latency (ms) | 243.40 ± 23.86 | 249.80 ± 25.01 | 0.178 |
| Lower Limb NRS baseline | 5.74 ± 2.35 | 6.19 ± 2.77 | 0.514 |
| Lower Limb DNIC N2–P2 amplitude (µV) | 19.52 ± 9.22 | 20.81 ± 7.49 | 0.903 |
| Lower Limb DNIC N2–P2 latency (ms) | 247.00 ± 27.34 | 248.41 ± 20.18 | 0.089 |
| Lower Limb NRS DNIC | 5.43 ± 2.38 | 5.45 ± 2.72 | 0.813 |
| Lower Limb post N2–P2 amplitude (µV) | 22.91 ± 15.27 | 18.16 ± 7.08 | 0.266 |
| Lower Limb post N2–P2 latency (ms) | 239.91 ± 26.73 | 244.41 ± 20.23 | 0.188 |
| Lower Limb NRS post | 5.67 ± 2.27 | 6.20 ± 2.61 | 0.253 |
| DNIC/baseline ratio | 0.87 ± 0.23 | 0.83 ± 0.15 | 0.638 |
| Right index finger sensory threshold | 2.48 ± 1.00 | 2.04 ± 0.85 | 0.031* |
| Finger stimulus intensity | 3.73 ± 1.51 | 3.08 ± 1.27 | 0.027* |
| Blink sensory threshold | 1.56 ± 0.33 | 1.68 ± 0.48 | 0.329 |
| Blink stimulus intensity | 15.57 ± 3.26 | 16.78 ± 4.78 | 0.329 |
| Ipsilateral R2 latency (ms) | 30.36 ± 3.37 | 31.05 ± 3.27 | 0.374 |
| Ipsilateral R2 area (ms·mV) | 1.06 ± 0.54 | 1.23 ± 0.66 | 0.242 |
| Contralateral R2 latency (ms) | 31.76 ± 3.13 | 32.67 ± 3.42 | 0.486 |
| Contralateral R2 area (ms·mV) | 0.95 ± 0.56 | 0.82 ± 0.55 | 0.224 |
| Mean R2 area (ms·mV) | 1.01 ± 0.47 | 0.94 ± 0.55 | 0.396 |

Legend: SD = standard deviation; LEPs = laser-evoked potentials; DNIC = diffuse noxious inhibitory control; NRS = numerical rating scale; µV = microvolt; ms = milliseconds; *, p < 0.05.

**Supplementary table 9–** Comparison of brain activity during fMRI tasks before and after rehabilitation in FMD patients.

| **fMRI MOTOR-TASK** | | | | | | |
| --- | --- | --- | --- | --- | --- | --- |
| **T1 > T0** | | | | | | |
| **Ke** | **T** | **BA** | **x** | **y** | **z** | **Area** |
| 14 | 3.79 |  | 10 | -34 | 54 | Right paracentral |
| **fMRI DUAL-TASK** | | | | | | |
| **T0 > T1** | | | | | | |
| **Ke** | **T** | **BA** | **x** | **y** | **z** | **Area** |
| 53 | 4.43 |  | 50 | 20 | 0 | Right inferior frontal opercularis/insula |
| **T1 > T0** | | | | | | |
| 18 | 4.11 |  | 10 | -58 | -48 | Right cerebellum IX |

X, Y and Z coordinates refer to the Montreal Neurological Institute (MNI) space. Table reports results of the paired t-test performed in SPM (p < 0.001). Only clusters greater than 10 voxels are reported.

fMRI motor-task consisted of antiphase feet dorsiflexion; fMRI dual-task consisted in the same feet movement while counting back by threes starting from 100. **Abbreviations:** BA = Brodmann Area; FMD = functional movement disorders; Ke = cluster extension; T0 = baseline (pre rehabilitation); T1 = post rehabilitation.

Supplementary Table 10. Availability of data across assessment domains at baseline and follow-up.

| **Domain** | **Participants (n/N)** | **Percentage (%)** |
| --- | --- | --- |
| Demographic variables | 34/34 | 100.0 |
| Psychometric variables | 34/34 | 100.0 |
| Sway parameters | 32/34 | 94.1 |
| Gait parameters | 25/34 | 73.5 |
| Laser-evoked potentials | 25/34 | 73.5 |
| Blink | 31/34 | 91.2 |

**Note.** Percentages were calculated based on the total study sample (*N* = 34). Missing data reflects participants for whom the corresponding assessment was not available or could not be analyzed due to technical issues or data quality criteria.
